# Assessment of *delirium* using the PRE-DELIRIC model in an intensive care unit in Argentina

**DOI:** 10.5935/0103-507X.20180010

**Published:** 2018

**Authors:** Fernando Ariel Sosa, Javier Roberti, Margarita Tovar Franco, María Mercedes Kleinert, Agustina Risso Patrón, Javier Osatnik

**Affiliations:** 1 Intensive Care Unit, Hospital Alemán - Buenos Aires, Argentina.; 2 Fundación para la Investigación y la Asistencia de la Enfermedad Renal - Buenos Aires, Argentina.

**Keywords:** *Delirium*/epidemiology, PRE-DELIRIC, CAM-ICU, Psychiatric status rating scales, Risk factors, Intensive care units

## Abstract

**Objective:**

To describe the incidence of and risk factors for *delirium* in the intensive care unit of a tertiary care teaching hospital in Argentina and to conduct the first non-European study exploring the performance of the PREdiction of DELIRium in ICu patients (PRE-DELIRIC) model.

**Methods:**

Prospective observational study in a 20-bed intensive care unit of a tertiary care teaching hospital in Buenos Aires, Argentina. The PRE-DELIRIC model was applied to 178 consecutive patients within 24 hours of admission to the intensive care unit; *delirium* was assessed with the Confusion Assessment Method for the Intensive Care Unit (CAM-ICU).

**Results:**

The mean age was 64.3 ± 17.9 years. The median time of stay in the intensive care unit was 6 (range, 2 - 56) days. Of the total number of patients, 49/178 (27.5%) developed *delirium*, defined as a positive CAM-ICU assessment, during their stay in the intensive care unit. Patients in the *delirium* group were significantly older and had a significantly higher Acute Physiological and Chronic Health Evaluation II (APACHE II) score. The mortality rate in the intensive care unit was 14.6%; no significant difference was observed between the two groups. Predictive factors for the development of *delirium* were increased age, prolonged intensive care unit stay, and opioid use. The area under the curve for the PRE-DELIRIC model was 0.83 (95%CI; 0.77 - 0.90).

**Conclusions:**

The observed incidence of *delirium* highlights the importance of this problem in the intensive care unit setting. In this first study conducted outside Europe, PRE-DELIRIC accurately predicted the development of *delirium*.

## INTRODUCTION

*Delirium*, a disturbance of consciousness with an acute onset and a variable course of impaired cognitive functioning, is common in patients admitted to the intensive care unit (ICU). Its incidence in this setting ranges from 16% to 80% depending on the population studied and diagnostic criteria used.^(1-5)^ Among the factors associated with *delirium* are unplanned extubation and catheter removal, nosocomial pneumonia, reintubation, the prolonged use of mechanically assisted ventilation, extended hospital stay, and long-term cognitive impairment.^(3,6-9)^ In routine practice, healthcare staff typically do not diagnose *delirium* in patients who present with the condition.^(3,10-12)^ However, the appropriate management of sedation and *delirium* can impact the outcome of ICU patients.^(10)^

Among the recommended methods for the diagnosis and assessment of *delirium* is the Confusion Assessment Method for the Intensive Care Unit (CAM-ICU).^(3)^ An increasing number of studies report that the risk of developing *delirium* depends on a complex interplay of factors.^(13)^ In ICU patients, the ability to predict *delirium* may help reduce its incidence, duration, and severity. The PREdiction of DELIRium in ICu (PRE-DELIRIC) model was recently developed for this purpose.^(14-16)^ Few studies have examined the incidence of *delirium* and its risk factors in the Argentinean population, and no studies have used PRE-DELIRIC to study Latin American populations.^(17)^

In this study, we investigated the incidence of and risk factors for *delirium* in the ICU of a tertiary care teaching hospital in Argentina and evaluated the performance of the PRE-DELIRIC model in this population.

## METHODS

The study was approved by the Ethics Committee of *Hospital Alemán* and was performed in accordance with international and national ethical standards and the guidelines of the Argentine National Administration of Drugs, Food, and Medical Technology (ANMAT). The study complied with Argentine Act 25326/Habeas Data. This was an observational, prospective cohort study performed in a 20-bed ICU of a tertiary care teaching hospital in Buenos Aires, Argentina, between 1 August 2016 and 30 January 2017.

This ICU is equipped for multi-organ support and has a nurse-patient ratio of 1:2. During the study period, all consecutive patients who were admitted to the ICU for ≥ 48 hours, were ≥ 18 years of age, and had a Richmond agitation and sedation scale (RASS) score between -2 and +4 were included in the study. Patients who had been treated with antipsychotic drugs within the previous 10 days, had a history of dementia, were suffering from acute alcohol withdrawal syndrome, had *delirium* or serious auditory or visual disorders before ICU admission, were unable to understand the Spanish or English languages, were severely mentally disabled, suffered from a terminal illness, or were < 18 years old were excluded. All personal information of the participants of this descriptive study remained anonymous and confidential.

The following information was collected upon admission: sex, date of admission, category of admission, diagnosis, description of previous and current use of sedatives or antipsychotic drugs, other medication used, Acute Physiological and Chronic Health Evaluation II (APACHE II) score, presence of invasive procedures, monitoring data, and type of organ support. Each patient's level of arousal was evaluated using the RASS score, which rates the level of agitation/sedation on a 10-point scale ranging from -5 (unarousable, not responsive to voice or physical stimulation) to +4 (combative). In addition, metabolic acidosis, urea concentration, the presence of infection, and coma status were assessed. Blood pressure, oxygen saturation, and electrocardiogram were continuously monitored.

The PRE-DELIRIC score was determined upon admission to the ICU. The PRE-DELIRIC model, developed and validated for ICU patients, assesses 10 risk factors for *delirium* that are readily observable within the first 24 hours following ICU admission.^(14-16)^ Because PRE-DELIRIC is a static model, it does not account for improvement or deterioration in health, but rather the change in the probability of *delirium* development.^(14-16)^ The following predictors in the PRE-DELIRIC model were obtained within the first 24 hours after ICU admission: age, APACHE II score, coma, urgent admission status (unplanned ICU admission), admission category (surgical, medical, trauma, or neurology/neurosurgical), infection status, sedative use, morphine use (three dosage groups), urea level, and metabolic acidosis.^(14)^ At our center, remifentanil is used instead of morphine; thus, the dosages were converted using a standard table. Acute renal failure was defined as the sudden decrease (over 48 hours) in renal function, as an increase in absolute serum creatinine of at least 26.5µmol/L (0.3mg/dL) or as a percentage increase in serum creatinine ≥ 50%; multiorgan failure was defined as the failure of ≥ 2 organs; and acute respiratory failure was defined as hypoxemia (partial pressure of oxygen - PaO_2_ < 60mmHg) with or without hypercapnia (partial pressure of carbon dioxide - PaCO_2_ > 50mmHg.

The presence of *delirium* was assessed using the CAM-ICU score, which was developed for evaluating four characteristics of *delirium* in critically ill, intubated patients: acute onset or fluctuating course of *delirium*, inattention, disorganized thinking, and altered level of consciousness.^(3,18)^ The Spanish version of the CAM-ICU has been validated. Two trained physicians performed the CAM-ICU evaluation once daily, in the morning, for each patient who met the inclusion criteria. In the case of discrepancies, a third ICU physician intervened. Further assessments were performed during the day if professionals detected disturbances in conscience, psychomotor behavior, emotion, mood, sensorium, and the sleep-wake cycle.

Comparisons were performed using Student's *t*-test, the Wilcoxon rank-sum test, Pearson's χ^2^ test, or Fisher's exact test as appropriate. A multivariate logistic regression model was used. Sensitivity, specificity, and the area under the receiver operating characteristic curve (AUC) with 95% confidence interval (CI) were calculated for PRE-DELIRIC scores. A p-value < 0.05 was considered to indicate statistical significance. All analyses were carried out using Stata v14 (StataCorp, College Station, TX). Categorical variables are expressed as frequencies and percentages, and continuous variables are given as the means and standard deviations or as medians with ranges.

## RESULTS

**Figure 1:**
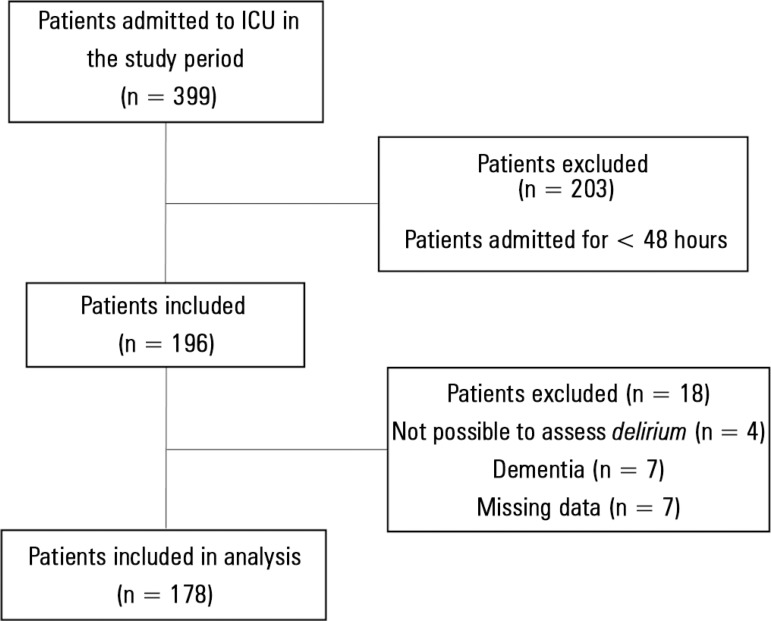
Flow diagram of patient inclusion.

We analyzed data from 178 patients admitted to the ICU. The flow diagram of patient inclusion is presented in figure 1, and the characteristics of the patients are shown in table 1. Of the 178 patients included in the study, 49 (27.5%) developed *delirium*, defined as a positive CAM-ICU assessment, during their ICU stay. Patients in the *Delirium* group (74.3 ± 9.4 years old) were significantly (p < 0.001) older than patients in the Non-*delirium* group (60.5 ± 18.8 years); patients in the former group also had a significantly higher APACHE II score (19.3±8.8 *versus* 12.6 ± 8.2, p < 0.005). The median time from admission to a positive CAM-ICU assessment was 5 (range, 1 - 44) days.

**Table 1:** Characteristics and outcomes of patients admitted to the intensive care unit

Characteristics	No *delirium* N = 129	*Delirium* N = 49	Total N = 178	p value
Age (years)	60.9 ± 18.4	74.4 ± 9.4	64.6 ± 17.5	0.000
Female	60 (45.1)	21 (42)	81 (44.3)	0.706
Stay in ICU (days)	5 (1 - 43)	13 (3 - 56)	6 (0 - 56)	0.000
PRE-DELIRIC score	0.48 ± 0.27	0.82 ± 0.22	0.57 ± 0.30	0.000
APACHE II score	12.5 ± 8.1	19.4 ± 8.7	14.4 ± 8.8	0.000
Reason for admission to ICU				
General ward	56 (43.4)	22 (44.9)	78 (43.8)	0.858
Surgery	53 (41.1)	17 (34.7)	70 (39.3)	0.436
Emergency department	20 (15.5)	10 (20.4)	30 (16.9)	0.435
Comorbidities				
COPD	13 (10.1)	10 (20.4)	23 (12.9)	0.066
Hepatobiliary disease/cirrhosis	4 (3.0)	1 (2.0)	5 (2.7)	1.000
Diabetes	11 (8.5)	2 (4.1)	13 (7.3)	0.309
Heart disease	13 (10.2)	5 (10.2)	18 (10.1)	0.587
Immunosuppression	17 (13.2)	4 (8.2)	21 (11.8)	0.354
Outcomes				
In-hospital death	15 (11.6)	11 (22.5)	26 (14.6)	0.068
Mechanical ventilation	23 (17.8)	26 (53.1)	49 (27.5)	0.000
Opioids (remifentanil)	67 (51.9)	39 (79.6)	106 (59.6)	0.001
Vasoactive agents	27 (20.9)	26 (53.1)	53 (29.8)	0.000
Sepsis	29 (22.5)	25 (51.0)	54 (30.3)	0.000
Multi-organ failure	8 (6.3.3)	11 (22.5)	19 (10.8)	0.005
Acute respiratory failure	33 (25.6)	24 (49.0)	57 (32.0)	0.003
Glucose < 80 or > 100mg/dL	5 (3.9)	2 (4.1)	7 (3.9)	1.000

ICU - intensive care unit; PRE-DELIRIC - PREdiction of DELIRium in ICu; APACHE II - Acute Physiology and Chronic Health Evaluation II; COPD - chronic obstructive pulmonary disease. Values are expressed as the mean ± standard deviation, n (%) or median (range).

The mortality rate among the ICU patients was 14.6%; no significant difference was observed between the two groups, although the incidence was higher in the *Delirium* group. Patients in this group also had a significantly higher rate of sepsis (25 [51.0%] *versus* 29 [22.5%]) and multi-organ failure (11 [22.5%] *versus* 8 [6.3%]) during the ICU stay than did patients in the Non-*delirium* group. The use of vasoactive agents and opioids was also significantly higher in the DG. Predictive factors for the development of *delirium* were older age, an additional day in the ICU, opioid use, and kidney failure (Table 2).

**Table 2:** Predictive factors for a positive Confusion Assessment Method for the Intensive Care Unit assessment

Variable	OR	SE	p value	95%CI
Age	1.07	0.020	0.000	1.03 -1.11
Stay in ICU (days)	1.09	0.025	0.000	1.05 - 1.14
Use of opioids	4.32	2.14	0.003	1.64 - 11.38
Kidney failure	2.88	1.61	0.059	0.96 - 8.62

OR - odds ratio; SE - standard error; 95%CI - 95% confidence interval; ICU - intensive care unit.

**Figure 2:**
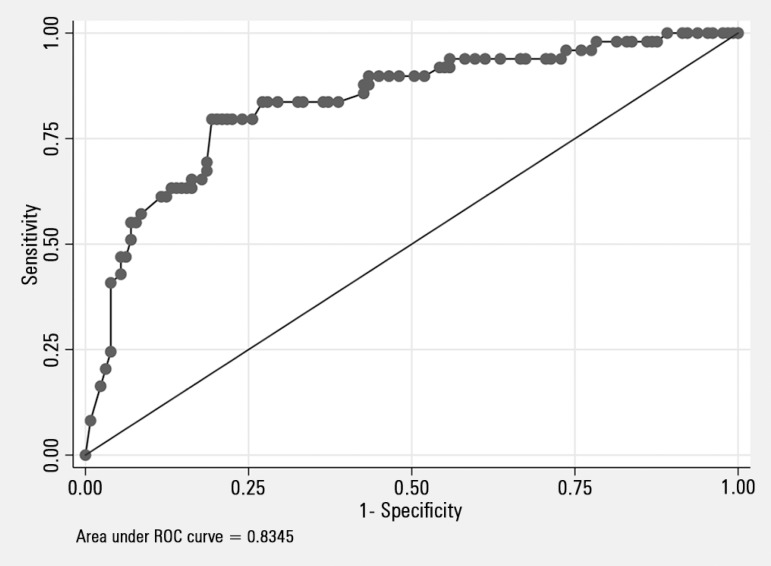
Receiver operating characteristic curve for the PREdiction of DELIRium in ICu model for predicting the development of *delirium* in the intensive care unit. ROC - receiver operating characteristic.

The discriminative power of the PRE-DELIRIC model for predicting *delirium* was determined based on an AUC of 0.84 (95%CI; 0.77 - 0.91). Figure 2 shows the AUC of the PRE-DELIRIC model. The different cutoff values are presented in table 3. For a PRE-DELIRIC score of 76%, the sensitivity for predicting the development of *delirium* was 80%, and the specificity was 79.70%.

**Table 3:** Area under the receiver operating characteristic curve cutoff points for the PREdiction of DELIRium in ICu model

Cutoff point (%)	Sensitivity( %)	Specificity (%)	Patients correctly classified (%)	LR+	LR-
20	98.00	16.54	38.80	1.1742	0.1209
50	90.00	54.89	64.48	1.9950	0.1822
60	84.00	65.41	70.49	2.4287	0.2446
70	80.00	77.44	78.14	3.5467	0.2583
76	80.00	79.70	79.78	3.9407	0.2509
80	66.00	82.71	78.14	3.8165	0.4111

LR+ - positive likelihood ratio; LR- - negative likelihood ratio.

## DISCUSSION

The 27% of ICU patients in this study who developed *delirium* did not exhibit significantly higher in-hospital mortality than the patients who did not develop *delirium*. The PRE-DELIRIC model predicted the development of *delirium* in our hospital setting.

Depending on the patient population and ICU type, the incidence of *delirium* reported in the literature varies significantly, ranging from 16% to 80%.^(3,17)^ The incidence of *delirium* in our study agrees with the results of an international study from Latin America that included Argentina.^(19)^ In other Argentine studies, the incidence of *delirium* was 43.3% in elderly hospitalized patients and 10.8% in adult patients admitted to the general ward.^(20,21)^

The predisposing and precipitating factors identified in our study are in accordance with previous reports.^(11,13,22,23)^ In fact, an association between age and *delirium* has often been described, thereby establishing *delirium* as a frequent complication in older ICU patients.^(2,11,13,24-26)^ An independent association between *delirium* and long-term mortality has been detected in critically ill patients and in those with severe pneumonia,^(25,27-29)^ but a counterfactual analysis showed that *delirium* prolongs the ICU stay but does not cause death in critically ill patients.^(9)^ Thus, the relationship between *delirium* and mortality remains unclear.

The purpose of the PRE-DELIRIC model is to identify patients at high risk for developing *delirium* within the first 24 hours of their ICU stay and therefore accelerate the initiation of preventive measures in this group.^(14)^ The PRE-DELIRIC model was developed in the Netherlands and is based on 10 risk factors. In a previous study, this model had a higher AUC than did prediction of *delirium* by attending caregivers (0.84 *versus* 0.59, respectively); our findings are in agreement with this result.^(14)^ Following the validation of the model in other European countries, its discriminative power was confirmed, and its calibration was optimized.^(15)^ Although one previous study reported an AUC of 0.77 for the PRE-DELIRIC model, the authors warned that its predictive value in other populations was unknown.^(15)^ However, in a prospective study encompassing seven countries, an AUC of 0.76 was reported.^(16)^ More recently, the model was applied to a Scottish cohort with a high prevalence of substance misuse, in which it predicted the development of *delirium*, length of ICU stay, and mortality at an early stage.^(30)^ The model demonstrated an acceptable predictive value and an AUC similar or better than that identified in previous studies in European ICUs. Our study is the first to assess the performance of the PRE-DELIRIC model outside Europe.

Our study had several limitations: its observational nature, the relatively small number of patients, the short follow-up period, and the fact that the duration of *delirium* was not recorded or correlated with outcomes or model performance.

Moreover, an important bias of the study was that partially due to the limited human resources at our hospital, *delirium* was assessed only in patients who exhibited signs of hyperactive *delirium* after the morning evaluation; this assessment criterion could have resulted in under-diagnosis.

The identification of risk factors for *delirium* could aid the development of preventive strategies.^(13,31,32)^ The rate of *delirium* in our ICU patients was 27%, which is in accordance with that in comparable populations. Our results also confirm the predictive value of the PRE-DELIRIC model and suggest that its use can contribute to the implementation of strategies to prevent or attenuate *delirium*.

## CONCLUSION

The incidence of *delirium* that we found highlights the importance of this problem in the intensive care unit setting. In this first study conducted outside Europe, PRE-DELIRIC accurately predicted the development of *delirium*.
